# Tandem Isotope Therapy with ^225^Ac- and ^177^Lu-PSMA-617 in a Murine Model of Prostate Cancer

**DOI:** 10.2967/jnumed.123.265433

**Published:** 2023-11

**Authors:** Catherine Meyer, Andreea Stuparu, Katharina Lueckerath, Jeremie Calais, Johannes Czernin, Roger Slavik, Magnus Dahlbom

**Affiliations:** 1 Ahmanson Translational Theranostics Division, Department of Molecular and Medical Pharmacology, David Geffen School of Medicine, UCLA, Los Angeles, California; and; 2 Clinic for Nuclear Medicine, University Hospital Essen, Essen, Germany

**Keywords:** PSMA-617, ^177^Lu, ^225^Ac, mouse model, prostate cancer, RLT

## Abstract

Radionuclide therapy targeting prostate-specific membrane antigen (PSMA) is a promising option for metastatic castration-resistant prostate cancer. Clinical experience using ^177^Lu or ^225^Ac has demonstrated encouraging treatment responses; however, responses are not durable. Dual-isotope combinations, or “tandem” approaches, may improve tolerability while retaining a high tumor dose. In this study, we directly compared α- versus β-particle treatment, as well as a combination thereof, at different stages of disease in a murine model of disseminated prostate cancer. **Methods:** First, to determine comparable injected activities from ^177^Lu- and ^225^Ac-PSMA-617, ex vivo biodistribution studies were performed at 5 time points after treatment of C4-2 subcutaneous tumor–bearing NSG mice. To establish a more representative model of metastatic prostate cancer, NSG mice were inoculated with luciferase-expressing C4-2 cells in the left ventricle, leading to disseminated visceral and bone lesions. At either 3 or 5 wk after inoculation, the mice were treated with equivalent tumor dose–depositing activities of ^177^Lu- or ^225^Ac-PSMA-617 alone or in combination (35 MBq of ^177^Lu, 40 kBq of ^225^Ac, or 17 MBq of ^177^Lu + 20 kBq ^225^Ac; 10/group). Disease burden was assessed by weekly bioluminescence imaging. Treatment efficacy was evaluated using whole-body tumor burden and overall survival. **Results:** The ex vivo biodistribution studies revealed that 35 MBq of ^177^Lu and 40 kBq of ^225^Ac yield equivalent absorbed tumor doses in a subcutaneous C4-2 model. The disease burden of mice treated at 3 wk after inoculation (microscopic disease) with ^177^Lu was not significantly different from that of untreated mice. However, ^225^Ac-PSMA-617 both as a single agent and in combination with ^177^Lu (17 MBq of ^177^Lu + 20 kBq of ^225^Ac) were associated with significant whole-body tumor growth retardation and survival benefit (overall survival, 8.3 wk for nontreatment, 9.4 wk for ^177^Lu, 15.3 wk for ^225^Ac alone, and 14.1 wk for tandem therapy). When treated at 5 wk after inoculation (macroscopic disease), all treatment groups showed retarded tumor growth and improved survival, with no significant differences between ^225^Ac alone and administration of half the ^225^Ac activity in tandem with ^177^Lu (overall survival, 7.9 wk for nontreatment, 10.3 wk for ^177^Lu, 14.6 wk for ^225^Ac alone, and 13.2 wk for tandem therapy). **Conclusion:** Treatment of a disseminated model of prostate cancer with simultaneous ^225^Ac- and ^177^Lu-PSMA-617 results in significantly decreased tumor growth compared with ^177^Lu, which was ineffective as a single agent against microscopic lesions. Mice treated later in the disease progression and bearing macroscopic, millimeter-sized lesions experienced significant tumor growth retardation and survival benefit in both monoisotopic and tandem regimens of ^177^Lu and ^225^Ac. Although the greatest benefits were observed with the single agent ^225^Ac, the tandem arm experienced no significant difference in disease burden or survival benefit, suggesting that the reduced activity of ^225^Ac was adequately compensated in the tandem arm. The superior therapeutic efficacy of ^225^Ac in this model suggests a preference for α-emitters alone, or possibly in combination, in the microscopic disease setting.

## Visual Abstract

**Figure:**
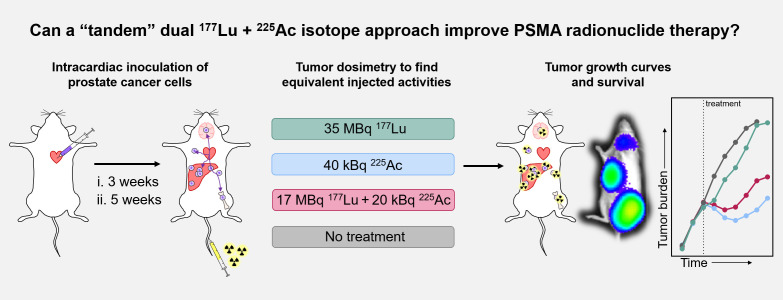

Radionuclide therapy targeting the prostate-specific membrane antigen (PSMA) protein is a promising option for patients with metastatic castration-resistant prostate cancer. The most commonly used therapeutic isotope is ^177^Lu, a medium-energy β-emitter (0.5 MeV) with a 6.7-d half-life (1). Response rates to ^177^Lu-PSMA-617 (as measured by >50% decline in serum biomarker prostate-specific antigen) have varied from 30% to 70% across treatment studies and patient cohorts (2–7). With a tissue penetration range of less than 2 mm and the ensuing cross-fire effect, ^177^Lu β-particles are best suited for treating lesions of a certain size (i.e., nonmicroscopic) (8–10). Although treatment with ^177^Lu-PSMA-617 is largely well tolerated, with a favorable dosimetry profile (11–13), in the setting of diffuse bone marrow infiltration, treatment with β-emitters may be limited by hematologic toxicity due to the irradiation of the surrounding healthy bone marrow tissues (10). ^225^Ac, an α-emitter with a 9.9-d half-life, has emerged as an alternative isotope with favorable therapeutic decay properties. α-particles are of interest in radionuclide therapy because of higher energy deposition over a much shorter tissue penetration range, causing more dense ionizations and localized dose profiles than for β-particles (14–16). The higher energy of ^225^Ac α-particles over a shorter tissue range (<0.1 mm) leads to a linear energy transfer on the order of 100 keV/μm (9*,*^17^–^19^). Decay schemes for ^177^Lu and ^225^Ac are shown in Figure 1.

**FIGURE 1.**
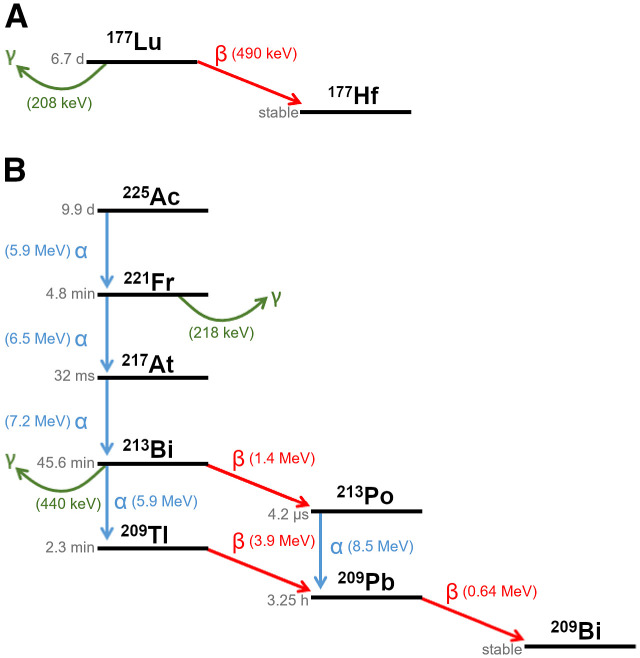
(A) ^177^Lu decay scheme. (B) ^225^Ac decay scheme.

Although fewer clinical studies have been conducted with ^225^Ac-PSMA-617, studies so far have reported biochemical response rates (>50% decline in prostate-specific antigen) ranging from 25% to over 90%, though patient cohorts, prior treatments, and treatment settings have varied widely (20–23). Additionally, α-particle therapy may be favored in the treatment of microscopic metastatic disease and bone marrow infiltration because of the shorter range of α-radiation (24–27). However, the same radiobiologic features that make ^225^Ac attractive against tumors also present a tradeoff at the expense of higher-grade toxicities (22). The most significant adverse effect of α-particle PSMA-targeted radionuclide therapy with small molecules (i.e., PSMA-617, PSMA-I&T) is xerostomia, making the salivary glands a key dose-limiting organ (28). Although the accumulation of PSMA ligands in the salivary glands is still not well understood, it is believed to be the result of both nonspecific (non–PSMA-related) and specific (PSMA-related) uptake mechanisms of PSMA ligands (29–31). Although xerostomia from ^177^Lu therapy is often temporary and reversible, with ^225^Ac there is a greater incidence of xerostomia, which can significantly diminish patient quality of life and lead to treatment discontinuation (22*,*^32^*,*^33^).

Overall, clinical experience using ^177^Lu or ^225^Ac has shown encouraging treatment responses; however, the responses have not been durable. Given that salivary gland toxicity limits the injected activity of ^225^Ac, simply using higher ^225^Ac treatment activities to increase tumor dose delivery is not an option. Dual-isotope combinations, or “tandem” approaches, may provide the benefits of both ^177^Lu and ^225^Ac to improve treatment tolerability while retaining high tumor dose delivery (10*,*^34^). Early clinical studies on both ^177^Lu-naïve patients and those who progressed after ^177^Lu-PSMA have shown that augmentation of ^177^Lu-PSMA therapy with a boost of ^225^Ac is an effective option with a more favorable side effects profile (35–37). No incidents of grade 3 or higher xerostomia were reported in these studies. A similar approach has been tested in peptide receptor radionuclide therapy against neuroendocrine tumors using tandem ^177^Lu/^90^Y-DOTATATE, combining 2 β-particle–emitting isotopes with different energies and tissue penetration ranges (38*,*^39^). In the preclinical setting, tandem ^177^Lu/^90^Y-DOTATATE demonstrated an antitumor effect superior to that of either monotherapy alone (40). For PSMA-targeted therapies, clinical data are still limited by small patient cohort sizes, and to date, there have been no systematic preclinical investigations into the efficacy of dual-isotope combinations. In this work, we sought to directly compare α- versus β-particle PSMA radionuclide therapy, as well as combinations of the two, in a mouse model of prostate cancer. Our hypothesis was that the emitted particle pathlength can impact the radiation dose delivery, especially to microscopic disease. The objective was to compare the treatment efficacy of a scaled combination of ^225^Ac and ^177^Lu to single-isotope treatments, as measured by longitudinal tumor control and survival. First, we conducted an ex vivo γ-counting biodistribution and tumor dosimetry study to determine injected activities of ^177^Lu and ^225^Ac that yield comparable tumor doses. We then treated mice bearing disseminated prostate cancer lesions at 2 different stages of disease with ^177^Lu- and ^225^Ac-PSMA-617 as single agents, or in combination, to compare therapeutic efficacy and survival.

## MATERIALS AND METHODS

### Cell Culture and Animal Studies

In all studies, the human-derived, PSMA-expressing prostate cancer tumor cell line C4-2 was used (courtesy of Dr. George Thalmann, Inselspital Bern, Switzerland). Cells were maintained in RPMI 1640 medium supplemented with 10% fetal bovine serum (Omega Scientific) and grown at 37°C and 5% CO_2_. Cells were monitored for *Mycoplasma* contamination using the Venor GeM *Mycoplasma* detection kit (Sigma Aldrich) and authenticated by short tandem repeat sequencing (Laragen). The parental cells were engineered to express firefly luciferase (C4-2-luc) to allow luciferase-mediated bioluminescence imaging to monitor tumor burden, as previously described (41).

All animal studies were approved by the UCLA Animal Research Committee (approval 2005-090). The mice were housed under pathogen-free conditions with food and water ad libitum and a 12 h–12 h light–dark cycle. Veterinary staff and investigators observed the mice daily to ensure animal welfare.

### Radiochemistry

PSMA-617 precursor was obtained from ABX Advanced Biochemical Compounds. ^177^Lu was obtained from Spectron MRC, and ^225^Ac was supplied by the U.S. Department of Energy’s Isotope Program within the Office of Science. Radiolabeling was performed as previously described with molar activities of 84 GBq/μmol and 130 MBq/μmol for ^177^Lu- and ^225^Ac-PSMA-617, respectively (41*,*^42^).

### Biodistribution and Tumor Dosimetry of ^177^Lu- and ^225^Ac-PSMA-617

Immunodeficient, 6- to 8-wk-old NOD SCID γ (NSG; The Jackson Laboratory) male mice were inoculated subcutaneously with 5 × 10^6^ C4-2 cells in 100 μL of Matrigel (Corning) into the shoulder region (50 mice). After 3 wk, when the tumors reached about 300 mm^3^ in volume, the mice were treated with either 30 MBq of ^177^Lu-PSMA-617 (25 mice) or 40 kBq of ^225^Ac-PSMA-617 (25 mice). The treatment activities were based on efficacious and well-tolerated activities in previous studies (43). The mice were euthanized at 1, 4, 24, 48, and 168 h after treatment (5 mice per time point for each nuclide). At the time of euthanasia, tumors and organs (including kidneys, liver, submandibular salivary glands, and intestines) were collected for ex vivo γ-counting for activity quantification (^177^Lu energy window, 189–231 keV; ^225^Ac energy window, 170–260 keV for ^221^Fr daughter detection; Cobra II Auto-Gamma; Packard Instrument Co.). Actinium samples were counted after 24 h when secular equilibrium was reached (44). The multiple *t* test method with Welch correction was used for biodistribution statistical comparisons (statistical significance set to ≤0.05).

We estimated tumor self-doses (ignoring cross-dose contributions from neighboring organs) by first curve-fitting and integrating the tumor time–activity curves (NUKFIT Software) (45). The total number of disintegrations was multiplied by dose constants to yield tumor doses for ^177^Lu and ^225^Ac (5.934 × 10^−1^ and 2.838 × 10^−3^ Gy⋅g/[μCi⋅h], respectively). Dose constants are derived from nuclear data for energy released per disintegration of each radionuclide, ignoring contributions with a decay yield of less than 1% (1*,*^46^). In this case of self-dose calculation, it is assumed that all radiation has an absorption fraction of 1.0 and that all disintegrations measured in the tumor deposit all energy in the tumor. We could thereby estimate the injected activities of ^177^Lu- and ^225^Ac-PSMA-617 that yield approximately equal tumor doses for subsequent studies directly comparing single- versus dual-isotope approaches.

### Tandem ^177^Lu/^225^Ac Therapy

All subsequent therapy studies were investigated in a mouse model of advanced metastatic prostate cancer. NSG mice were inoculated with C4-2-luc cells in the left ventricle, leading to disseminated visceral and bone lesions, as previously described (80 mice) (41). The mice were treated at 2 different stages of disease—either 3 or 5 wk after inoculation with equivalent tumor dose-depositing activities of ^177^Lu- or ^225^Ac-PSMA-617 or in scaled combination. Three weeks after inoculation, the disseminated lesions are approximately 200 μm in size, increasing to millimeter scale by 5 wk, as previously characterized (41). The treatment activities were determined by the tumor biodistribution and dose-finding study as previously described. The treatment groups were as follows: 35 MBq of ^177^Lu-PSMA-617, 40 kBq of ^225^Ac-PSMA-617, a mixture of 17 MBq of ^177^Lu-PSMA-617 plus 20 kBq of ^225^Ac-PSMA-617, or untreated (10 mice per group per treatment time, intravenous administration; the chosen activities are justified in the Results section). The tandem-isotope treatment regimen was designed to halve the respective doses of ^225^Ac and ^177^Lu in combination.

Disease burden was assessed by weekly luciferase-mediated bioluminescence imaging (IVIS Lumina III; Perkin Elmer), and the mice were followed for overall survival. The mice were euthanized when their overall condition showed signs of deteriorating health based on the body conditioning score (47). Therapeutic efficacy data regarding tumor burden, as measured by whole-body radiance over time (Living Image; Perkin Elmer), were analyzed with 1-way ANOVA with Bonferroni adjustment using GraphPad Prism 8. The log-rank (Mantel–Cox) test was used for survival analysis.

## RESULTS

### Biodistribution and Tumor Dosimetry of ^177^Lu- and ^225^Ac-PSMA-617

**FIGURE 2.**
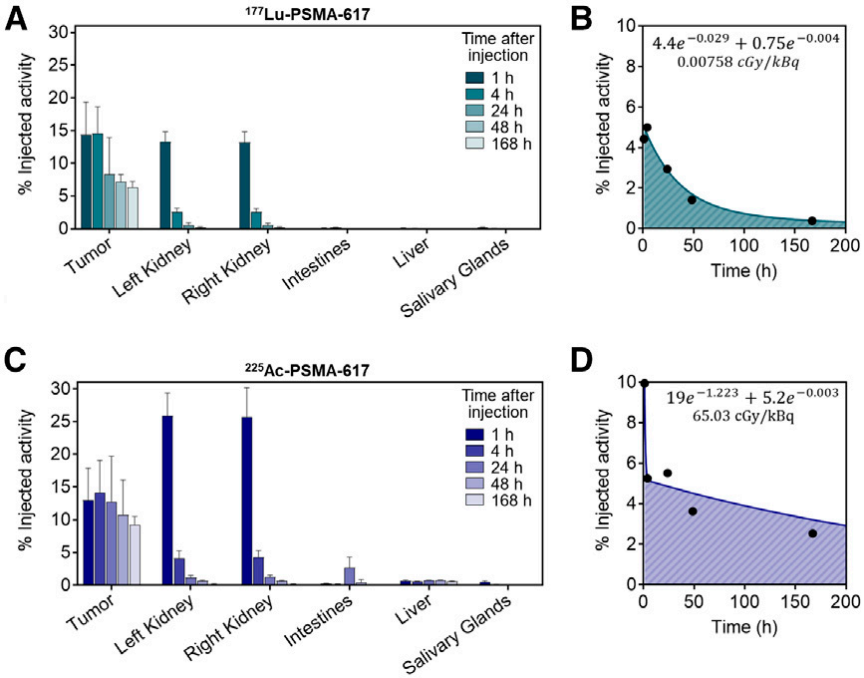
Ex vivo biodistribution and tumor time–activity curves for ^177^Lu- and ^225^Ac-PSMA-617. (A) Mean ± SD percentage injected activity per gram of tissue for mice treated with 30 MBq of ^177^Lu-PSMA-617 (5 mice per time point). Tabulated values and additional organs are available in supplemental materials. (B) Mean percentage injected activity of ^177^Lu-PSMA-617 in tumors over time used for curve-fitting and dosimetry. Best-fit parameters are shown. (C) Mean ± SD percentage injected activity per gram of tissue for mice treated with 40 kBq of ^225^Ac-PSMA-617 (5 mice per time point). Tabulated values and additional organs are available in supplemental materials. (D) Mean percentage injected activity of ^225^Ac-PSMA-617 in tumors over time used for curve-fitting and dosimetry. Best-fit parameters are shown.

The ex vivo biodistribution of ^177^Lu- and ^225^Ac-PSMA-617 in subcutaneous tumors and organs is shown in Figure 2. Tabulated biodistribution values for tumors and organs are available in Supplemental Tables 1 and 2 (supplemental materials are available at http://jnm.snmjournals.org). Kidney uptake at 1 h after injection was significantly greater for ^225^Ac than for ^177^Lu (25.9% ± 3.5% and 13.3% ± 1.6% injected activity/g, *P* < 0.0005), but no statistical difference was observed at later time points. Both ^177^Lu- and ^225^Ac-PSMA-617 rapidly localized to the tumor, with similar uptake (14.4% ± 4.1% and 14.1% ± 4.9% injected activity/g, respectively) at 4 h after intravenous injection (not statistically significant; *P* = 0.89). Tumor uptake peaked at 4 h after injection, and by 168 h (7 d), tumor uptake was significantly greater in ^225^Ac-treated tumors than in ^177^Lu-treated tumors (9.3% ± 1.3% vs. 6.3% ± 0.9% injected activity/g, *P* < 0.004, 5 mice per radionuclide). Tumor time–activity uptake curves in percentage injected activity were used for curve-fitting in NUKFIT (Figs. 2B and 2D). NUKFIT software selected the best curve-fitting model in both cases to be in the following form: $A_{1}e^{\text{−}\lambda_{1}t}+A_{2}e^{\text{−}\lambda_{2}t},\;$where $\lambda_{1}$ and $\lambda_{2}$ represent the fitted decay constants. The best-fit curve parameters are shown in Figure 2. The resultant cumulated activities were multiplied by the respective dose constants and normalized by injected activity and average tumor masses. The tumor-absorbed doses for ^177^Lu- and ^225^Ac-PSMA-617 in the subcutaneous C4-2 model were 0.00758 and 65.03 cGy/kBq, respectively, or roughly 850 times greater for ^225^Ac than for ^177^Lu. Therefore, from this study we decided to use 35 MBq of ^177^Lu and 40 kBq of ^225^Ac to yield similar absorbed tumor doses in the therapeutic efficacy studies. Previous work demonstrated that 40 kBq of ^225^Ac-PSMA-617 is well tolerated (43). For the tandem treatment arm, we chose to halve the activity of each isotope in combination (17 MBq of ^177^Lu + 20 kBq of ^225^Ac) to test a more tolerable activity regimen.

### Tandem ^177^Lu/^225^Ac Therapy

**FIGURE 3.**
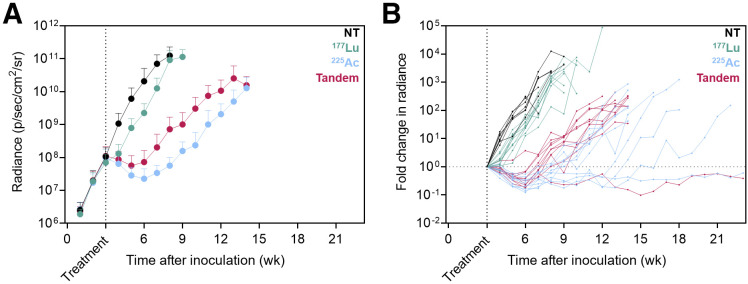
Therapeutic efficacy for mice treated 3 wk after inoculation with ^177^Lu/^225^Ac-PSMA-617 or in tandem. (A) Mean ± SD whole-body radiance over time (10 mice per group). Data are shown for time points with 5 or more remaining mice. (B) Individual mouse fold change in radiance over time relative to disease burden at time of treatment. NT = no treatment.

**FIGURE 4.**
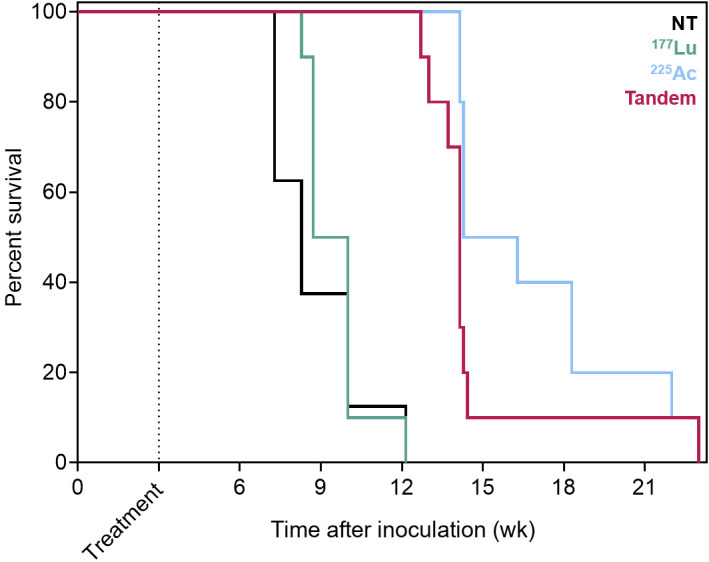
Survival curves for mice treated 3 wk after inoculation with ^177^Lu/^225^Ac-PSMA-617 or in tandem. Median overall survival increased from 8.3 to 9.4 wk for ^177^Lu-PSMA-617, 14.1 wk for tandem therapy, and 15.3 wk for ^225^Ac-PSMA-617 alone (*P* = 0.337 for NT vs. ^177^Lu, *P* < 0.0001 for tandem vs. ^177^Lu; *P* < 0.0001 for ^225^Ac vs. ^177^Lu; *P* = 0.108 for ^225^Ac vs. tandem). NT = no treatment.

Whole-body bioluminescence imaging radiance over time and mouse survival curves for treatment 3 wk after inoculation are shown in Figures 3 and 4, respectively. Notably, the disease burden of mice treated at this earlier stage of disease with ^177^Lu-PSMA-617 was not significantly different from that of untreated mice at any time point (*P* = 0.932). However, ^225^Ac-PSMA-617 both as a single agent and in combination with ^177^Lu-PSMA-617 was associated with significant tumor growth retardation (*P* = 0.009 for tandem vs. ^177^Lu; *P* = 0.0084 for ^225^Ac vs. ^177^Lu; *P* > 0.999 for tandem vs. ^225^Ac measured 5 wk after treatment). If mice were left untreated, median survival was 8.3 wk. When mice were treated with 35 MBq of ^177^Lu-PSMA-617, no significant survival benefit was observed (median survival, 9.4 wk; *P* = 0.337). However, when treated with one of the ^225^Ac regimens, the median survival increased to 14.1 wk for the tandem approach and 15.3 wk for ^225^Ac-PSMA-617 alone (*P* < 0.0001 for tandem vs. ^177^Lu; *P* < 0.0001 for ^225^Ac vs. ^177^Lu; *P* = 0.108 for ^225^Ac vs. tandem).

**FIGURE 5.**
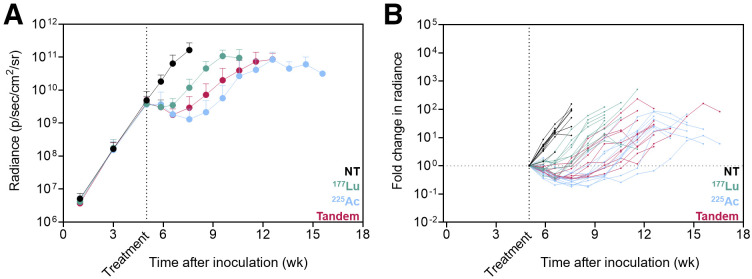
Therapeutic efficacy for mice treated 5 wk after inoculation with ^177^Lu/^225^Ac-PSMA-617 or in tandem. (A) Mean ± SD whole-body radiance over time (10 mice per group). Data are shown for time points with 5 or more remaining mice. (B) Individual mouse fold change in radiance over time relative to disease burden at time of treatment. NT = no treatment.

**FIGURE 6.**
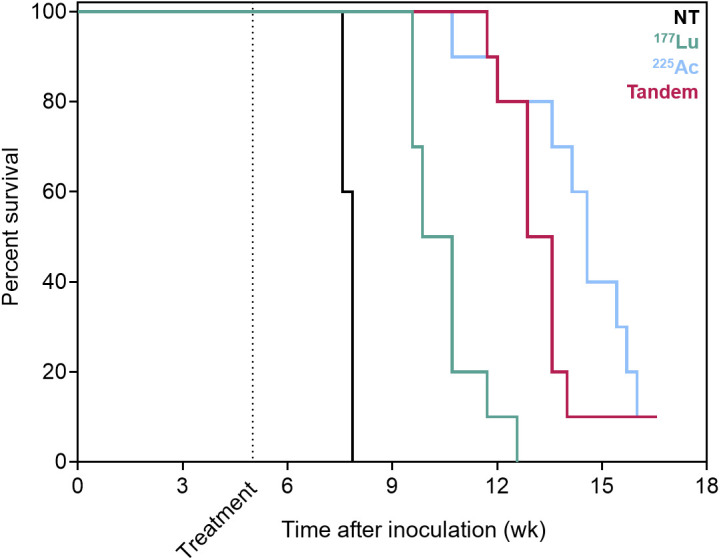
Survival curves for mice treated 5 wk after inoculation with ^177^Lu/^225^Ac-PSMA-617 or in tandem. Median overall survival increased from 7.9 to 10.3 wk for ^177^Lu-PSMA-617, 13.2 wk for tandem therapy, and 14.6 wk for ^225^Ac-PSMA-617 alone (*P* < 0.0001 for NT vs. ^177^Lu, *P* < 0.0001 for tandem vs. ^177^Lu; *P* < 0.0001 for ^225^Ac vs. ^177^Lu; *P* = 0.171 for ^225^Ac vs. tandem). NT = no treatment.

When mice were treated at a later time point of macroscopic disease (5 wk after inoculation), all treatment groups showed retarded tumor growth relative to untreated mice (Fig. 5). However, the greatest benefits were observed with ^225^Ac-PSMA-617 monotherapy and tandem approaches (*P* < 0.0001 for ^225^Ac vs. ^177^Lu; *P* < 0.0001 for tandem vs. ^177^Lu measured 5 wk after treatment). Median overall survival increased from 7.9 wk (untreated) to 10.3 wk for ^177^Lu-PSMA-617, 13.2 wk for tandem therapy, and 14.6 wk for ^225^Ac-PSMA-617 alone (*P* < 0.0001 for NT vs. ^177^Lu; *P* < 0.0001 for tandem vs. ^177^Lu; *P* < 0.0001 for ^225^Ac vs. ^177^Lu) (Fig. 6). There were no significant differences in whole-body disease burden or survival benefit conferred between ^225^Ac alone and halving the ^225^Ac activity in tandem with ^177^Lu (*P* = 0.171 for ^225^Ac vs. tandem survival and *P* > 0.999 for ^225^Ac vs. tandem whole-body radiance 5 wk after treatment).

## DISCUSSION

To our knowledge, this was the first study reporting on the efficacy of ^177^Lu/^225^Ac-PSMA tandem-isotope combinations in a mouse model of prostate cancer. In this work, we sought to compare the treatment efficacy of the same tumor dose delivered by 3 different radiation mechanisms: β-particles (^177^Lu), α-particles (^225^Ac), or both (^177^Lu + ^225^Ac). To do so, we first determined comparable injected activities to yield comparable tumor doses by conducting a full ex vivo biodistribution study using both ^177^Lu-PSMA-617 and ^225^Ac-PSMA-617. It is important to note that the applied injected activities were chosen so as to expose the cancerous lesions to comparable absorbed radiation doses, without adjustment for the effectiveness of the type of radiation. Interestingly, there was no significant difference in peak tumor uptake of ^177^Lu- or ^225^Ac-PSMA-617 at 4 h after injection; however, increased tumor retention was observed at all subsequent time points for mice treated with ^225^Ac-PSMA-617 compared with ^177^Lu-PSMA-617. Although normal-organ biodistribution was not the primary objective of this report, all biodistribution data are available in the supplemental materials. In addition to the kidneys, liver, salivary glands, and intestines included in this report, uptake data for the following additional organs are available: blood, heart, lungs, spleen, stomach (with contents), prostate, testes, muscle, femur (with and without bone marrow), bone marrow, and brain.

In this work, we challenged a tandem-isotope approach against an advanced model of widespread disease to compare the dynamics of tumor control and overall survival. To more objectively compare the treatment arms against one another, we first determined injected activities for ^177^Lu and ^225^Ac that expose the tumors to similar absorbed doses in the subcutaneous C4-2 model. We then designed the tandem arm to be a combination of half of each respective single-agent activity. The tandem-isotope approach was tested with the intracardiac inoculation model of C4-2 cells, at 2 different stages of disease progression with different volumes of lesions. Although subcutaneous tumors allow for straightforward uptake quantification, and therefore interrogation into absorbed doses, they fail to recapitulate the metastatic state. Dosimetry was not feasible in the intracardiac inoculation model because the lesions are not easily isolated, especially in microscopic stages. Translating dosimetry findings from subcutaneous models is one approach toward standardizing the applied activity when the tumor burden is distributed throughout the mouse and direct dosimetry is not possible. Furthermore, we sought to investigate the performance of tandem therapy in a model that more faithfully represents the setting in which treatment with α-particles (alone or in combination) would actually be clinically warranted.

The intracardiac inoculation model is an aggressive prostate cancer model in which mice, when left untreated, succumb to extensive disseminated disease warranting euthanasia by 8 wk after inoculation (Figs. 4 and 6). This model has previously been shown to be sensitive to treatment with ^225^Ac-PSMA-617 early in the disease, but this work is the first effort, to our knowledge, at challenging at a very advanced stage (5 wk after inoculation) (41). By treating at 2 different stages of disease, we can also seek to answer what size lesions most benefit from treatment with ^177^Lu or tandem approaches. At the onset of the earlier treatment time investigated (3 wk after inoculation), the disseminated lesions are approximately 200 μm in size, increasing to millimeter scale by 5 wk, as previously characterized (41). When given at 3 wk, ^177^Lu-PSMA-617 as a single agent did not significantly improve tumor control or survival relative to untreated mice (Fig. 4). However, when lesions were millimeters in size at the time of treatment, ^177^Lu-PSMA-617 retarded tumor growth and the mice conferred a significant survival advantage. Failure of ^177^Lu-PSMA-617 against micron-scale lesions in the earlier treatment setting can be explained by a mismatch between the target lesion size and the pathlength of the therapeutic radiation. With a mean tissue range of 600 μm (9), β-particles from ^177^Lu travel a distance that exceeds the lesion size at the time of early treatment (3 wk). This results in a loss of specificity to the targeted lesions and attenuates tumor response, as confirmed in this study.

Given the tissue range of less than 0.1 mm, α-particles from ^225^Ac yield dense ionizing paths with little to no cross-fire effect. For treatment at both stages of disease, mice treated with ^225^Ac-PSMA-617 as a single agent survived the longest and experienced the best tumor control (despite not reaching statistically significant differences at all time points). Even when challenged with lesions on the millimeter scale, the single agent ^225^Ac-PSMA-617 outperformed ^177^Lu-PSMA-617. Interestingly, halving the ^225^Ac activity in tandem with ^177^Lu did not significantly increase the whole-body disease burden as measured 5 wk after treatment (Figs. 3A and 5A). In this model, the comparable tumor control and survival between the full ^225^Ac dose and the tandem dose regimen suggest that a reduced administered activity of ^225^Ac could be adequately compensated with ^177^Lu without significantly sacrificing effectiveness. The superior therapeutic efficacy of ^225^Ac in the microscopic setting studied in this work suggests a preference for α-emitters alone, or possibly in combination, for treatment of microscopic or minimal residual disease.

This work compares therapeutic isotopes using a fixed activity prescription for the tandem-isotope treatment arm. However, in the few published studies describing tandem treatment approaches of ^177^Lu- and ^225^Ac-PSMA-617 in the clinical setting, the applied injected activities have been heterogeneous. The mean reported activity of ^177^Lu-PSMA-617 has ranged from 6 to 6.7 GBq, whereas the mean activity of ^225^Ac-PSMA-617 has ranged from 2.7 to 4 MBq (35*,*^37^). Another study reported a median ^177^Lu-PSMA-617 activity of 6.9 GBq (ranging from 5.0 to 11.6 GBq) and 5.3 MBq (ranging from 1.5 to 7.9 MBq) for ^225^Ac-PSMA-617 (36). This wide range in applied activities precludes interpretation of a recommended activity scheme, and the heterogeneity underscores that, in practice, the treatment activities should reflect the individual patient condition. Additional studies are thereby needed to establish maximum tolerated doses in tandem-isotope schemes. One such prospective study examining the combination of ^177^Lu-PSMA-I&T and ^223^Ra (AlphaBet trial NCT05383079) is under way using a fixed 7.4 GBq of ^177^Lu-PSMA administration followed by escalating activities of ^223^Ra (48). Furthermore, prior treatments, including the cumulative injected activity of ^177^Lu-PSMA-617 as a monotherapy if applicable, should also be considered in the activity prescription. Yet another factor to consider in the design of tandem-isotope treatments is the timing of the relative administrations. Although our preclinical work investigated the simultaneous administration of ^177^Lu and ^225^Ac, tandem treatment on consecutive days or in the first few days of each cycle also warrants investigation. Further work is required to understand the optimal conditions under which to prescribe tandem-isotope approaches, including how to adapt the treatment activities to reflect individual patient tumor burden, metastatic extent, and prior treatments.

One limitation of the mouse models used in these studies is the inability to recapitulate critical organ uptake as seen in patients (i.e., in the salivary glands and kidneys). In the subcutaneous tumor model, kidney uptake was higher with ^225^Ac than with ^177^Lu at 1 h after injection; however, no significant difference was observed beyond the initial uptake phase. Clinically, kidney and salivary gland equivalent doses from ^177^Lu-PSMA-617 were reported in one study as 0.39 and 0.36–0.58 Sv/GBq, respectively (13). For ^225^Ac-PSMA-617, one dosimetry report calculated kidney and salivary gland doses to be 0.74 and 2.33 Sv/MBq, respectively (assuming a relative biologic effectiveness factor of 5 for ^225^Ac) (49). However, our biodistribution study revealed no significant differences in submandibular gland uptake between ^177^Lu- and ^225^Ac-treated mice, and peak uptake was less than 0.5% of injected activity at all measured time points (Fig. 2). Given this inherent limitation in the translatability of salivary gland toxicity, it was not within the scope of this study to assess the preclinical feasibility of tandem-isotope treatment to improve the toxicity profile.

## CONCLUSION

Treatment of a microscopic model of prostate cancer with 40 kBq of ^225^Ac-PSMA-617 or 20 kBq of ^225^Ac in tandem with 17 MBq of ^177^Lu resulted in significantly decreased tumor growth compared with ^177^Lu, which was ineffective as a single agent against microscopic lesions, likely because of a mismatch of particle pathlength and lesion size. Mice treated later (when lesions were millimeter scale in size) experienced significant tumor growth retardation and survival benefit in both monotherapy and tandem regimes of ^177^Lu- and ^225^Ac-PSMA therapy. However, the greatest benefits were observed with ^225^Ac-PSMA-617 as a single agent and in tandem approaches. Further work is needed to identify the disease patterns and settings that most benefit from treatment with β-particles, α-particles, or both.

## DISCLOSURE

Katharina Lueckerath reports paid consulting activities for Sofie Biosciences/iTheranostics and funding from AMGEN outside the submitted work. Jeremie Calais reports prior consulting activities for Advanced Accelerator Applications, Blue Earth Diagnostics, Curium Pharma, GE Healthcare, EXINI, IBA RadioPharma, Janssen Pharmaceuticals, Lantheus, POINT Biopharma, Progenics, Radiomedix, and Telix Pharmaceuticals. No other potential conflict of interest relevant to this article was reported.

## Notes

KEY POINTS **QUESTION:** Does dual-isotope tandem ^177^Lu/^225^Ac-PSMA radionuclide therapy improve treatment outcomes in a mouse model of disseminated prostate cancer? **PERTINENT FINDINGS:** We determined equivalent tumor dose–depositing injected activities of ^177^Lu- and ^225^Ac-PSMA-617 and tested treatment as single agents versus in combination against different-sized lesions. ^225^Ac alone, and in tandem with ^177^Lu, was superior to ^177^Lu as a single agent against microscopic lesions. No significant difference was observed between the tandem and ^225^Ac treatment arms. **IMPLICATIONS FOR PATIENT CARE:** This work provides efficacy data for ^177^Lu- and ^225^Ac-PSMA-617 as single agents and in combination against lesions of micron to millimeter scale. These data can be used as a basis for further investigations into the disease conditions best suited for treatment with β-particles, α-particles, or both.
